# Conjugated Bile Acids Promote Lymphangiogenesis by Modulation of the Reactive Oxygen Species–p90RSK–Vascular Endothelial Growth Factor Receptor 3 Pathway

**DOI:** 10.3390/cells12040526

**Published:** 2023-02-06

**Authors:** Priyanka Banerjee, Subhashree Kumaravel, Sukanya Roy, Niyanshi Gaddam, Johnny Odeh, Kayla J. Bayless, Shannon Glaser, Sanjukta Chakraborty

**Affiliations:** 1 Department of Medical Physiology, Texas A&M Health Science Center, Bryan, TX 77807, USA; 2 Department of Molecular and Cellular Medicine, Texas A&M University Health Science Center, Bryan, TX 77807, USA

**Keywords:** bile acids, lymphatic endothelial cells, lymphangiogenesis, oxidative stress, growth factor signaling

## Abstract

Conjugated bile acids (BA) are significantly elevated in several liver pathologies and in the metastatic lymph node (LN). However, the effects of BAs on pathological lymphangiogenesis remains unknown. The current study explores the effects of BAs on lymphangiogenesis. BA levels were elevated in the LN and serum of Mdr2^−/−^ mice (model of sclerosing cholangitis) compared to control mice. Liver and LN tissue sections showed a clear expansion of the lymphatic network in Mdr2^−/−^ mice, indicating activated lymphangiogenic pathways. Human lymphatic endothelial cells (LECs) expressed BA receptors and a direct treatment with conjugated BAs enhanced invasion, migration, and tube formation. BAs also altered the LEC metabolism and upregulated key metabolic genes. Further, BAs induced the production of reactive oxygen species (ROS), that in turn phosphorylated the redox-sensitive kinase p90RSK, an essential regulator of endothelial cell dysfunction and oxidative stress. Activated p90RSK increased the SUMOylation of the Prox1 transcription factor and enhanced *VEGFR3* expression and 3-D LEC invasion. BA-induced ROS in the LECs, which led to increased levels of Yes-associated protein (YAP), a lymphangiogenesis regulator. The suppression of cellular YAP inhibited BA-induced VEGFR3 upregulation and lymphangiogenic mechanism. Overall, our data shows the expansion of the lymphatic network in presclerotic liver disease and establishes a novel mechanism whereby BAs promote lymphangiogenesis.

## 1. Introduction

Elevated bile acids (BAs) are associated with chronic inflammatory liver diseases such as cholestasis, alcoholic liver disease, steatosis, and liver cancers such as hepatocellular carcinoma and cholangiocarcinoma (CCA) [1]. BAs are amphipathic steroidal molecules that, under physiological conditions, aid lipid absorption, the triglyceride metabolism, and the regulation of energy homeostasis. BAs have differential binding affinities with receptors, including nuclear farnesoid X receptor (FXR), TGR5 (which is a G-protein-coupled receptor (GPCR), sphingosine-1-phosphate receptor 2 (S1PR2), pregnane X receptor (PXR), and vitamin D receptor (VDR) [2]. Elevated levels of conjugated BAs such as taurochenodeoxycholic acid (TCDCA) and taurocholic acid (TCA) have been found in patients’ sera from CCA patients and in several inflammatory liver pathologies [3,4,5]. Interestingly, high levels of BAs have been reported in metastatic tumor-draining lymph nodes [6]. Increased lymphangiogenesis is seen in several liver pathologies, including liver cancers [7] and cholestatic diseases [8], but the mechanisms mediating these cellular events remain elusive.

The liver has a dense network of lymphatic vessels and is the largest producer of lymph in the body [9]. Along with the regular functions such as body fluid homeostasis and the removal of protein, cholesterol, and immune infiltrates, lymphatic vessels play critical roles in the modulation of inflammatory and immune responses [7,10]. Lymphangiogenesis or the growth of new lymphatic vessels may be beneficial or detrimental in an organ or pathology-dependent manner and LECs are active participants in this process [11,12]. BAs are reported to increase oxidative stress in hepatocytes via the production of reactive oxygen species (ROS), creating an inflammatory environment [13,14]. Hepatic lymphatics are formed close to the portal triad, near the portal vein and bile duct, hence we hypothesized that, during the progression of liver disease, the lymphatics, especially LECs, are continuously exposed to high levels of pathological BAs. Despite the well-established role of BAs on several liver pathologies, their effect on LECs or the regulation of lymphangiogenesis has not been studied. The development and maintenance of the lymphatic vasculature, as well as pathological lymphangiogenesis, requires vascular endothelial growth factor receptor 3 (VEGFR3/VEGFC) signaling [15], and the maintenance of LECs fate is closely regulated by the homeodomain transcription factor Prospero homeobox protein 1 (Prox1) [16]. It has been shown that Prox1 is a target of a small ubiquitin-like modifier (SUMO-1) and SUMOylated Prox1 at K556 increases the transcription of VEGFR3 [17]. Pan et al. have also shown that the SUMOylation of Prox1 is regulated by the SUMO-specific protease 2 (sentrin/SUMO-specific protease 2 or SENP2) the deSUMOylation enzyme [17]. SENP2 is regulated by the redox-sensitive serine/threonine kinase p90 ribosomal S6 kinase (p90RSK) [18]. p90RSK is a member of the Src-ERK1/2 signaling pathway, activated by ROS [19]. The activated p90RSK can phosphorylate SENP2 at T368, and phosphorylated SENP2 lost its de-SUMOylation activity. Pathological BAs are known to produce ROS [20]; and based on this, we hypothesized that BA-induced ROS activates p90RSK-mediated Prox1 SUMOYlation and VEGFR3 transcription, which leads to increased lymphangiogenesis. A recent study showed that elevated level of ROS in hepatocellular carcinoma increased the expression of the Yes-associated protein 1 (YAP1), the effective transcription factors of the Hippo pathway [21]. It has also been shown that YAP signaling is essential for developing lymphatic vasculature in Zebrafish through the activation of VEGFC signaling in LECs [22]. Interestingly, elevated levels of BAs in hepatocellular carcinoma were reported to activate the YAP signaling [23]. Further, in LN metastatic tumors, high levels of bile acids found in the LN were reported to activate YAP via its dephosphorylation [6].

Hence, in this study, we investigated the effects of conjugated BAs on LEC phenotypes and lymphangiogenesis. We provide evidence for a novel mechanism showing that BAs induce the ROS-p90RSK-YAP-VEGFR3 axis to regulate oxidative stress response pathways and lymphangiogenesis.

## 2. Materials and Methods

### 2.1. Animal Model

All experiments were performed according to the guidelines, approval was obtained from the Texas A&M University (College Station, TX, USA) Institutional Biosafety Committee and TAMU IACUC, and they were in compliance with the ARRIVE guidelines. Friend virus B-type (FVB)/NJ (Control for Mdr2^−/−^ mice) and Mdr2^−/−^ mice (a mouse model of primary sclerosing cholangitis) (male, 25–30 gm, 6 to 12 weeks of age) were purchased from Jackson Laboratories (Bar Harbor, ME, USA) [24]. All mice were housed in a temperature-controlled environment, with 12:12-h light–dark cycles with access ad libitum to water and standard mouse chow. 

The measurements of BA from the mouse sera and LN: blood samples were collected from mice by a cardiac puncture to avoid any contamination, kept in tubes without anticoagulant treatment, and left at room temperature for 30 min. To isolate the sera, the blood samples were centrifuged at 2000× *g* for 10 min at 4 °C and the serum samples were collected for the measurement of the total bile acid level using the Mouse Total Bile Acid Assay kit (Cell BioLab Inc., San Diego, CA, USA). Mice liver LNs were collected from the mice after necropsy. The liver LN tissues were homogenized for 30–40 s, followed by centrifugation at 10,000× *g* for 10 min at 4 °C, and the supernatant were collected in fresh tubes. All the samples were 5-times diluted with deionized H_2_O. The standard curve was prepared as per the manufacturer’s instructions. Then, 20 µL of the samples and standards were added to the 96-well plate and 150 µL of assay reagent A was added to each well, followed by incubation at 37 °C for 5 min. All the samples were added in duplicate. After 5 min of incubation, 50 µL of assay reagent B was added to each well, mixed thoroughly, and then 50 µL of NADH reagent was added. The plate was incubated for 30 min at 37 °C. Finally, the total bile acid was measured colorimetrically (at a primary wavelength of 405 nm and a secondary wavelength 630 nm) using a microplate spectrophotometer following the manufacturer’s protocol. 

### 2.2. Cell Culture

Human dermal lymphatic endothelial cells (HLECs) from three different de-identified donors were purchased from Promocell (Heidelberg, Germany) and were maintained in complete endothelial basal media (EBM-MV2, Promocell, Heidelberg, Germany) at 37 °C with 5% CO_2_. The cell characteristics were verified by the expression of the HLEC specific markers, namely LYVE1, PROX1, and podoplanin (PDPN), as described previously [25]. The TGR5 specific inhibitor SBI-115(3-methylphenyl 5-chloro-2-(ethylsulfonyl)-4-pyrimidinecarboxylate) was purchased from Sigma Aldrich (St. Louis, MO, USA). The RSK inhibitor BI-D1870 was purchased from Selleckchem (Houston, TX, USA). The YAP inhibitor verteporfin was purchased from Cayman Chemical (Ann Arbor, MI, USA) and the N-Acetyl-L-cysteine (NAC), the specific ROS inhibitor, was purchased from Sigma Aldrich (St. Louis, MO, USA). For the inhibitor treatments, HLECs were pre-treated with SBI-115 (10 µM), BI-D1870 (1 µM), NAC (10 mM), or verteporfin (0.5 µM) 1 h prior to the BA treatment. The cell permeable TGR5 receptor agonist (phenoxypyrimidine carboxamide derivative) was purchased from Sigma Aldrich. For the RNA and protein isolation from BAs-treated HLECs, 50–150 µM of conjugated BAs were used to treat HLECs for 24 h. 

### 2.3. XTT Assay

The XTT Cell Proliferation Assay Kit from Trevigen (Gaithersburg, MD, USA) was used to measure cell proliferation following the manufacturer’s protocol. Briefly, 3 × 10^4^ HLEC cells were seeded onto a 96-well plate. The cells were treated with TCA and TCDCA (Sigma Aldrich) with varying concentrations of 50 µM, 100 µM, and 150 µM for 24 h. We selected that dose range as previous in vitro studies showed that at this dose range, conjugated BA increased cellular proliferation [26,27,28]. Then, 50 µL of XTT working solution was added to the wells and incubated at 37 °C with 5% CO_2_ for 6 h and the absorbance was measured at 490 nm, with a reference wavelength of 630–690 nm.

### 2.4. RNA Extraction and Real-Time PCR 

The total RNA was extracted from the cells treated as above using the PureLink™ RNA Mini Kit (Invitrogen, Waltham, MA, USA) following the manufacturer’s protocol. For RNA isolation from mice liver LNs, the tissues were rapidly snap frozen in liquid N_2_ immediately after their isolation and stored at −80 °C in a freezer until further processing. RNA isolation was done using a PureLink™ RNA Mini Kit (Thermo Fisher Scientific, Waltham, MA, USA) following the manufacturer’s protocol. Briefly, snap frozen tissues were homogenized with an appropriate volume of RNA lysis buffer provided with the kit and homogenized by rotor-stator, in a chilled, 4 mL round bottom RNase-free tube for 30–40 s, followed by centrifugation at 2600× *g* for 5 min at room temperature. The supernantant was carefully transferred to a fresh RNase-free tube and proceeded to subsequnt binding, washing, and elution steps following the manufacturer’s protocol. The RNA quality and quantity were measured using NanoDrop Technologies (NanoDrop Technologies, Wilmington, DE, USA) and, subsequently, cDNA was prepared using the Maxima H Minus cDNA Synthesis Kit from Life Technologies (Carlsbad, CA, USA). Real-time PCR was performed for the genes for chemokines, cytokines, cellular stress, markers for the lymphatic system, and lymphangiogenesis using the PowerUp™ SYBR™ Green Master Mix (Applied Biosystems, Foster City, CA, USA) in the real-time thermal cycler (ABI Prism 7900HT sequence detection system; Applied Biosystems) and each reaction was performed in triplicate. The data analysis was conducted using the 2^−ddCt^ method [29]. Ubiquitin/ribosomal protein L19 (*RPL19*) genes were used as the housekeeping genes for the data analysis. The sequences of the primers (Sigma Aldrich) used for the specific genes are listed in Table 1. Premade primers for *FXR*, *TGR5*, *VDR* were purchased from Sigma Aldrich. 

### 2.5. Protein Extraction and Western Blot Analysis

The protein samples for the Western blot analysis were prepared by lysing the cells using the M-PER protein extraction buffer (Thermo Fisher Scientific, Waltham, MA, USA) with 1X phosphatase inhibitor cocktail (PIC) (Cell Signaling Technology, Danvers, MA, USA) and 1X PMSF phenyl methylsulfonyl fluoride (Fluka, Buchs, Switzerland). The protein concentration was measured using the Pierce™ Rapid Gold BCA Protein Assay Kit (Thermo Fisher Scientific). The cell lysates were run in Bolt™ 4 to 12%, Bis-Tris, 1.0 mm, mini protein gel (Invitrogen, MA, USA) along with Spectra™ Multicolor Broad Range Protein Ladder (Invitrogen). They were transferred to a nitrocellulose membrane (Bio-Rad Laboratories, Hercules, CA, USA). The membranes were blocked in 5% milk, probed with the specific primary antibodies listed in Table 2, and finally in corresponding anti-rabbit or anti-mouse secondary antibodies. The protein bands were visualized using the chemiluminescence detection kit (Pierce, Thermo Fisher Scientific) and the images were captured in the ImageQuant™ LAS 4000 (Fujifilm, Tokyo, Japan). The densitometric analysis was performed using ImageJ software (Wayne Rasband, NIH, Bethesda, MD, USA). 

### 2.6. Immunoprecipitation and Detection of SUMOylation by Western Blot

The cells were lysed with the appropriate volume of Pierce™ IP Lysis Buffer (Thermo Fisher Scientific) supplemented with 1X PIC and 1X PMSF and 25 mM of N-ethylmaleimide (NEM) (Thermo Fisher Scientific). Lysates were placed on ice for 10 min and centrifuged at 4 °C for 15 min at 15,000× *g* to collect protein lysates. For immunoprecipitation, 500 µL of cell lysate (1 mg/mL) was diluted with the Pierce™ IP Lysis Buffer supplemented with PIC, PMSF, and NEM and incubated with PROX1 antibody (Cell Signaling Technology, Danvers, MA, USA) and as the control normal rabbit IgG (Cell Signaling Technology) overnight at 4 °C with rotation. After overnight incubation, 50 mL of protein A/G agarose beads were washed twice with the IP lysis buffer, were added to each sample, and incubated at room temperature for 2 h with gentle rotation, followed by elution with IgG Elution Buffer (ThermoScientific). As described above, the eluted samples were run in 12%, Bis-Tris, 1.0 mm, Mini Protein Gel. The PROX1 SUMOylation was detected by probing the blot with an anti-SUMO1 antibody (Cell Signaling Technology) [17]. 

### 2.7. Measurement of Total ROS and Mitochondrial ROS (mtROS) 

To measure the level of total cellular ROS and mtROS, HLECs were plated into a 96-well plate with the seeding density of 3 × 10^4^ cells/well. The cells were pretreated with a specific BA inhibitor, SBI-115 (10 mM), RSK specific inhibitor BI-D1870 (1 µM) (Selleckchem, TX, USA), and NAC (10 mM) 1 h before treatment with specific BAs, TCA, and TCDCA (Sigma Aldrich) (100 µM) for 45 min for the total ROS and 12 h for mtROS. Following the treatment, the cells in the 96-well plate were washed with warm Hank’s balanced salt solution with calcium and magnesium (HBSS) (Sigma Aldrich) and incubated with 2.5 µM of MitoSOX™ Red in HBSS (100 mL/well) (Thermo Fisher Scientific) for 10 min in the dark in a 5% CO_2_, 37 °C incubator and then washed three times with HBSS. The production of mtROS was measured by fluorescent plate reader at excitation/emission maxima of approximately 510/580 nm (Spectra Max i3, Molecular Devices, San Jose, CA, USA). For total cellular ROS measurement, after the treatment with 100 µM of TCA and TCDCA for 45 min, the cells were washed with warm 1X HBSS and incubated with 100 mL/well of the master mix, prepared as per the manufacturer’s instructions of the Fluorometric Intracellular ROS Kit (Sigma Aldrich), for 30 min in a 5% CO_2_, 37 °C incubator. After 30 min of incubation, the cells in the plate were washed twice with warm HBSS and the fluorescence intensity was measured at excitation/emission maxima of 540/570 nm. 

### 2.8. Seahorse Assay

HLECs were seeded into the Xfe96 plates at a density of 3 × 10^4^ in 5% FBS containing EGM-MV2 media per well, as per the manufacturer’s protocol, from the Seahorse XFp Cell Energy Phenotype Test Kit (Agilent Technologies, Santa Clara, CA, USA). They were incubated in a 5% CO_2_ atmosphere at 37 °C for 24 h. The data were recorded using Seahorse Xfe96 extracellular flux analyzer according to the manufacturer’s protocol. Three drugs, oligomycin (an ATP synthase inhibitor), rotenone, and antimycin A (Rot/A.A.) (inhibitor of respiratory complex I and II, respectively), were used to calculate the OCR and ECAR. Oligomycin (1.5 µM) and Rot/A.A. (0.5 µM) were used as per the manufacturer’s protocol. After 24 h, the media was changed to 5% FBS containing EGM and the cells were treated with TCA and TCDCA (100 µM) and incubated in a 5% CO_2_ atmosphere at 37 °C for 24 h. Following 24 h of treatment, the cell culture media was removed, except for 20 µL, and was replaced with the Agilent Seahorse XF Base Medium supplemented with 1 mM pyruvate, 2 mM glutamine, and 10 mM glucose, with the pH adjusted to 7.4, with 0.1 N NaOH. The cells were kept at 37 °C in a CO_2_-free incubator for 1 h. The basal oxygen consumption rate (OCR) and extracellular acidification rate (ECAR) were measured in the Xfe96 plate reader using Wave software version 2.1.6 (Agilent Technologies, Santa Clara, CA, USA), as per the manufacturer’s instructions. In this assay, the OCR values of the pre-oligomycin and post-Rot/A.A. injection are regarded as the maximal glycolytic capacity [30,31]. Beginning with an 18 min baseline, the baseline values for the OCR, ECAR, and metabolic potential were calculated based on the proportion of stressed OCR and stressed ECAR for 48 min. The normalization of the Seahorse results was performed using the CyQuant cell proliferation assay kit (Thermo Fisher Scientific) as described previously [25,31,32,33]. Data analysis was performed using Wave software version 2.1.6 (Agilent Technologies, Santa Clara, CA, USA) and GraphPad Prism Software version 9 (GraphPad Software, Inc., San Diego, CA, USA). 

### 2.9. siRNA Transfection

Two YAP1 siRNA sequences (siYAP1-1, siYAP1-2), siProx1 (Sigma Aldrich) and a fluorescein amidite (FAM) tagged negative control (siControl; negative Control), were purchased from Sigma Aldrich. HLECs were seeded in 30 mm dishes and allowed to grow until at 50–60% confluency. According to the manufacturer’s instructions, they were then transfected with 8μL of siPORTAmine (Thermo Fisher Scientific, Waltham, MA, USA) and each siRNA (50–150 nM). The HLECs were then treated with or without TCDCA or TCA (100 μM) for 24 h. The isolation and Western analyses of the total proteins from these samples were performed as mentioned above. The knockdown of YAP and Prox1 were confirmed by probing the blot with a total YAP antibody (Cell Signaling Technology) and real time PCR, respectively. 

### 2.10. Transwell Migration

Approximately 1 × 10^5^ HLECs, were allowed to migrate towards 5% FBS containing EGM-MV2 with or without 100 µM of TCDCA or TCA through collagen-coated transwell inserts with 8 µm pores with a membrane [34]. After 24 h, the inserts were removed. The cells inside the membrane were swabbed and the cells that migrated were fixed with methanol and stained with crystal violet. Images were taken with 10X objective using the Nikon Digital Sight microscope. The number of cells migrated was calculated using ImageJ software.

### 2.11. Invasion Assays 

HLECs invasion assays were performed as described previously [35]. Briefly, HLECs were allowed to form a monolayer on three-dimensional collagen matrices containing various growth factors and then treated with or without TCDCA or TCA (100 µM). To study the effect of TGR5 or p90RSK inhibition, HLECs were pretreated with SBI-115 or BI-D1870 for 1 h before the addition of TCA or TCDCA. The cells were allowed to invade for 24 h, fixed with 3% glutaraldehyde (Sigma Aldrich) in PBS, and stained with 0.1% toluidine blue (Sigma Aldrich) in 30% methanol. For image sprouting HLECs, toluidine blue-stained samples were cut and imaged from the side with 10X or 20X objectives using an Olympus microscope as previously described [35]. The number of sprouts formed per 0.25 mm^2^ field was quantified manually using an eyepiece fitted with a 10 × 10 ocular grid. 

### 2.12. Tube Formation Assay

Equal numbers of HLECs were seeded on top of Matrigel (BD Biosciences) in a 96-well plate and were treated with or without 100µM of conjugated BAs (TCDCA or TCA). For TGR5 or p90RSK inhibitor treatment, HLECs were pretreated with the SBI-115 or BI-D1870 for 1 h before adding bile acids. The tube formation was then monitored over 4 h, after which the tubes were stained with 2 µM Calcein, AM (Invitrogen, Thermo Fisher Scientific), and imaged under 4X objective using the Olympus fluorescent microscope. The total branching length was measured using ImageJ software.

### 2.13. Immunofluorescence (IF) and Immunohistochemistry (IHC)

HLECs were grown on coverslips and treated with or without TCDCA or TCA (100 µM) for 24 h. The cells were fixed with 4% paraformaldehyde, permeabilized with ice-cold methanol, and blocked with goat serum. The cells were incubated with total primary YAP (Novus Biologicals, Littleton, CO, USA) or β-Catenin (Santa Cruz Biotechnology, Dallas, TX, USA) antibodies for 2 h at room temperature. The cells were washed and then incubated with fluorescence-tagged secondary antibodies for 1 h at room temperature. After washing, the coverslips were mounted on slides and imaged with 40X objectives in a fluorescent microscope (Olympus). Liver and liver nodes were isolated from control (FVB) and Mdr2^−/−^ mice and embedded in the OCT (optimal cutting temperature) (TissueTek, Torrance, CA, USA) compound. The tissue sections from the liver and liver lymph node from the control FVB and Mdr2^−/−^ mice were cut at a thickness of 10 µm with a Leica CM 1860 (Leica, Heidelberg, Germany) cryostat machine at a core facility of the Department of Medical Physiology at the College of Medicine, Texas A&M University (College Station, TX, USA). These sections were stained with CK19 (MyBioSource, San Diego, CA, USA), LYVE-1 (R&D Systems, Minneapolis, MN, USA), and VEGFR3 (R&D Systems, Minneapolis, MN, USA) in 1:100 dilution. 

### 2.14. Statistical Analysis

All experiments were done at least in triplicate. The mean values of the experimental groups were compared using GraphPad Prism v9 software, following the statistical methods that included unpaired Student’s *t*-test during the analysis of two groups and a one- or two-way analysis of variance (ANOVA) for a comparison between more than two groups, followed by an appropriate post hoc test. The values were represented as the means ± SD and the group-wise difference was considered significant when the *p* value < 0.05. 

## 3. Results

### 3.1. The Mouse Model of Liver Cholestasis has a High Level of Lymphangiogenesis

**Figure 1:**
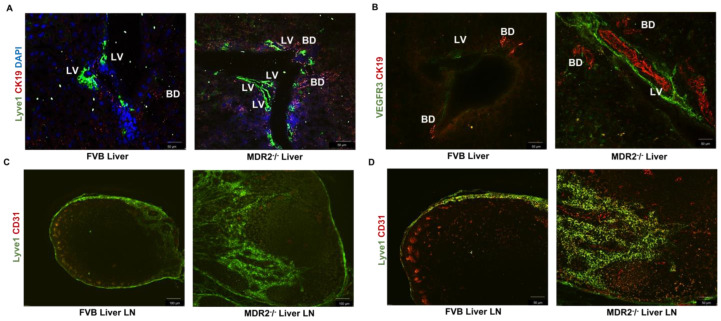
Mouse model of primary sclerosing cholangitis (Mdr2^−/−^) shows enhanced level of lymphangiogenesis in liver and liver lymph nodes (liver LN). Immunofluorescence images of liver tissue sections from Mdr2^−/−^ mice (**A**,**B**) taken at 20× magnification show high level of LYVE-1 expression (**A**) (green) and VEGFR3 (green) expression (**B**), indicating upregulated hepatic lymphatic infiltration. The cholangiocytes were stained with CK 19 (red). (**C**,**D**) Liver lymph node (liver LNs) images taken at 10× and 20× magnification from Mdr2^−/−^ mice showed increased LYVE-1(green) and CD31 (red) expression, indicating high nodal lymphatic infiltration. Scale bar is 50 μm and 100 μm.

Increased levels of lymphangiogenesis have been associated with inflammation and are well documented in liver cancers; however, increased lymphangiogenesis has been associated with cholestasis [7,36,37,38]. Thus, we first analyzed the lymphatic infiltration in the livers of Mdr2^−/−^ mice, a model for sclerosing cholangitis and pre-cancerous hepatocellular carcinoma that is characterized by significant inflammation and liver fibrosis [39]. The immunohistochemical analysis of the liver tissue sections and liver lymph node (LN) for detecting lymphangiogenesis was performed by staining for CK19, VEGFR3, and LYVE1. Compared to the control FVB mice, the Mdr2^−/−^ mice have significantly higher lymphatic vessel infiltration in the liver lymph node (Figure 1A–D). 

### 3.2. The Mouse Model of Liver Cholestasis has Enhanced Levels of Bas in Lymph Nodes and Serum

**Figure 2:**
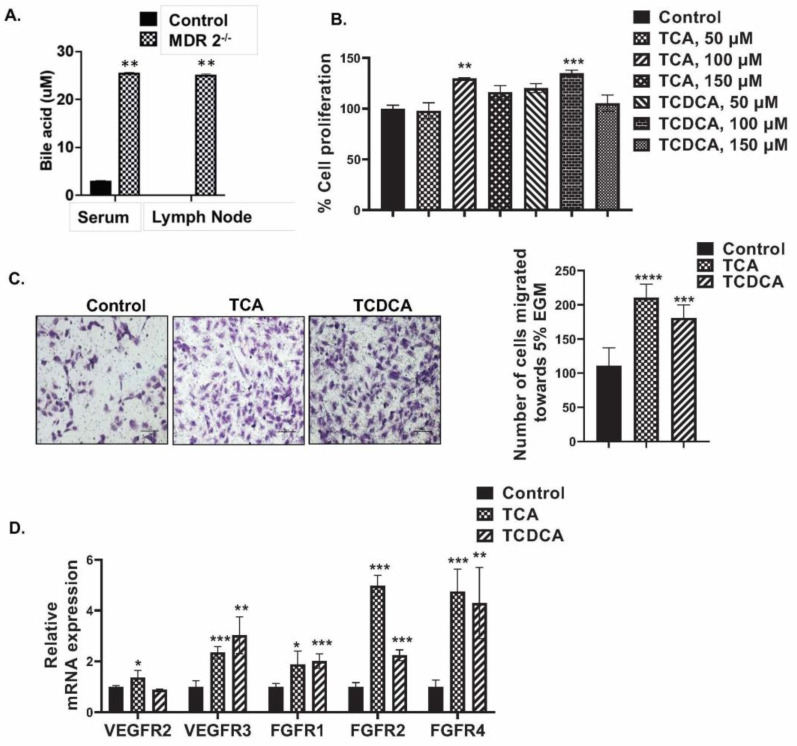
Elevated levels of BAs are found in lymph nodes of PSC mice and BAs induce activation of pro-lymphangiogenic mechanisms in vitro. (**A**) BA concentrations (in µM) in serum and lymph nodes of Mdr2^−/−^ mice. Values represent mean ± SD, *n* = 3. (**B**) Conjugated BAs induced HLEC proliferation was measured by XTT assay and data represented as % of cell proliferation. Values are represented as mean ± SD, *n* = 5 (**C**) TCDCA and TCA (100 µM)-induced migration of HLECs through the Transwell inserts towards 5% FBS containing EGM (5% EGM) at 24 h. (**D**) TCDCA and TCA (100 µM)-induced mRNA levels of lymphangiogenic growth factor receptors in 5% EGM for 24 h were measured by real time PCR assay. Values represent mean ± SD, *n* = 3. Statistical analysis was done by ANOVA followed by Fishers LSD. *, **, ***, and **** represents *p* ≤ 0.05, *p* ≤ 0.01, *p* ≤ 0.001, and *p* ≤ 0.0001, respectively, as compared to control.

Pathological BAs are elevated in cholestatic liver diseases, non-alcoholic fatty liver diseases, and liver cancer and significantly contribute to disease pathogenesis [40,41]. However, because the levels of BAs have not been measured in the liver lymph nodes in pathological conditions, we measured the BA levels in the serum and lymph nodes of Mdr2^−/−^ mice [41]. The BAs were significantly elevated (approximately 10-fold) in the serum and the liver lymph nodes of Mdr2^−/−^ mice compared to FVB control mice (Figure 2A). 

### 3.3. Conjugated BAs Enhance the Proliferation and Migration of HLECs 

To understand the effect of elevated conjugated BAs on HLECs, we analyzed the proliferation of HLECs treated with TCA and TCDCA (50 µM, 100 µM, and 150 µM) for 24 h. Both TCDCA and TCA at a 100 µM concentration significantly increased the proliferation of HLECs (*p* ≤ 0.05 compared to control) (Figure 2B). We also evaluated the effects of conjugated BAs on the HLEC migration. We found that both TCDCA and TCA significantly increased (*p* < 0.05) the migration of HLECs compared to the controls, indicating the increased migratory potential of cells treated with conjugated BAs (Figure 2C). 

### 3.4. Conjugated BA Increased the Expression of Lymphangiogenic Growth factor Receptors 

Further, we wanted to determine whether conjugated BAs affect the lymphangiogenic growth factor receptors, namely vascular endothelial growth factor receptors *VEGFR2* and *VEGFR3*, as well as fibroblast growth factor receptors *FGFR2* and *FGFR4*. We have found that TCA and TCDCA significantly increased the mRNA expression of *VEGFR3*, the receptor for VEGFC, and *FGFR1*, *FGFR2*, and *FGFR4* at a significant level (*p* < 0.05) (Figure 2D). These data indicate the ability of conjugated BAs to promote LEC proliferation and migration and induce the expression of the lymphangiogenic growth factor receptors in HLECs.

### 3.5. HLECs Express BA Receptors 

**Figure 3:**
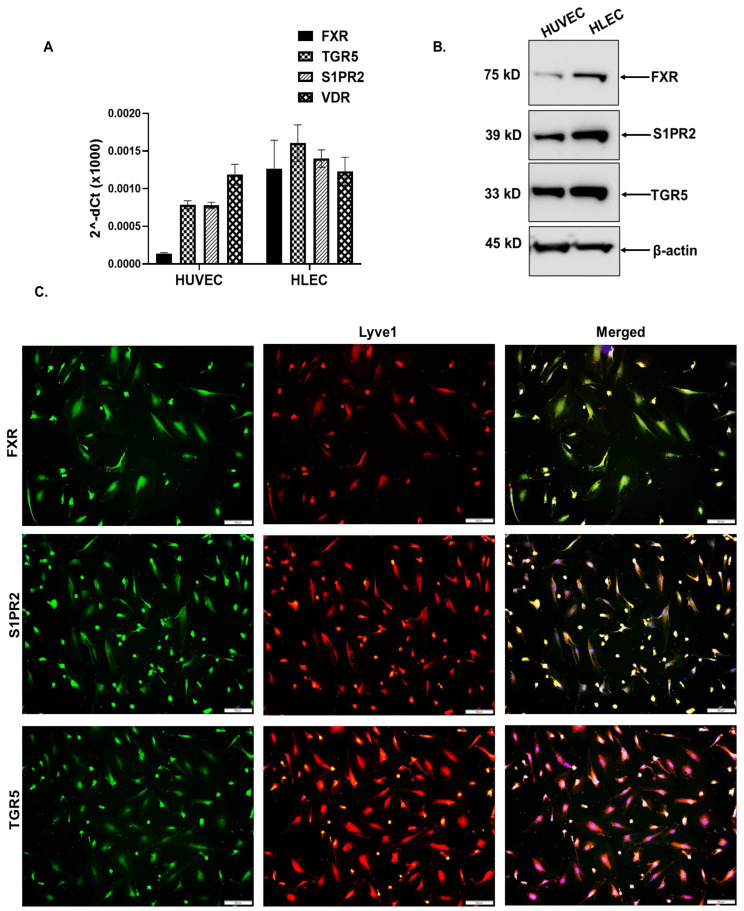
HLECs express the BA receptors FXR, S1PR2, TGR5, and VDR. (**A**) The basal level mRNA expression of the BA receptors, *FXR*, *S1PR2*, *TGR5*, and *VDR* were measured by qRT-PCR and the data were presented at 2^−dCt^. (**B**,**C**) The protein level expression was measured by Western blot (**B**) and immunofluorescence (**C**) with the specific antibodies. HLECs were stained with Lyve-1 (red) in immunofluorescence. Values represent mean ± SD, *n* = 3. Scale bar is 100 μm.

No study has carefully evaluated the expression of BA receptors on HLECs or its role in the modulation of the lymphangiogenesis sprouting of new lymphatic vessels. Hence, we first determined whether HLECs express BA receptors. The basal level of the BA specific cell surface receptors, sphingosine 1-phosphate receptor (*S1PR*) *2*, Takeda G-protein receptor 5 (*TGR5*), farnesoid X receptor (*FXR*), and vitamin D Receptor (*VDR*), were evaluated by a real-time PCR in HLECs. Among the four BA receptors, the basal level of the mRNA expression (2^^−dCt^) of the *TGR5* was higher compared to the others in the untreated HLEC and we also compared the expression in human umbilical vein endothelial cells (HUVEC), another endothelial cell line from a non-lymphatic origin which had a lower expression than that in HLECs (Figure 3A). We have also checked the protein level expression by Western blot and immunofluorescence (Figure 3B,C). In the subsequent experiments, we used the TGR5 antagonist SBI-115 (10 µM) to determine if the conjugated BA-mediated impact on HLECs were regulated through TGR5 receptor.

### 3.6. Conjugated BAs Alters the Cellular Metabolism and Activate the Redox Pathway in HLECs through TGR5 Receptors 

**Figure 4:**
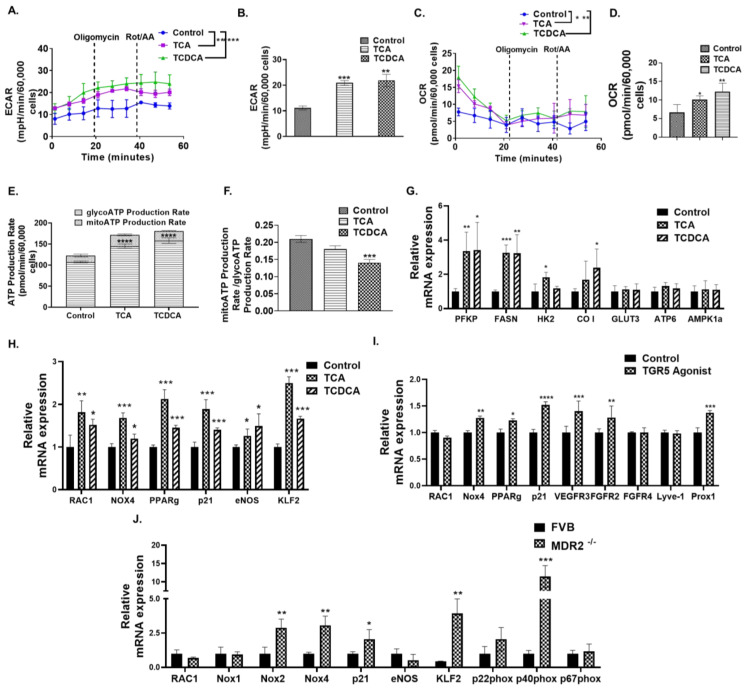
Conjugated BAs alters cellular metabolism and enhances expression of genes involved in the Re-dox pathway. (**A**–**F**) Conjugated BAs alter the cellular metabolism in HLECs. The extracellular acidification rate (ECAR) of control and conjugated BAs (TCA and TCDCA) (100 µM)-treated groups were measured by Seahorse XFp Cell Energy Phenotype Test Kit and plotted using the Wave software. The ECAR was represented at time points after sequential addition of oligomycin (1.5 µM) and Rot/A.A. (0.5 µM). The basal oxygen consumption rate (OCR) was significantly increased by conjugated BAs. (**E**,**F**) The comparison between glycolytic and mitochondrial ATP production rate in 5% EGM, TCA, and TCDCA (100 µM) treated HLECs showed that conjugated BAs (100 µM) increased the glycolytic ATP production rate in HLECs. (**G**) Conjugated BAs also increased the mRNA expression of the metabolic genes *PFKP*, *HK2*, *CO I*, and *FASN*. (**H**) Conjugated BAs induced the mRNA expression of the redox genes *RAC1*, *Nox4*, *PPARγ*, *p21CIP*, *eNOS*, and *KLF2* in HLECs after 24 h of treatment in 5% EGM. (**I**) The TGR5 agonist (2.5 nM) treatment for 24 h increased the mRNA expression of growth factor receptors and redox genes in HLECs. (**J**) The mRNA expression of the redox genes *p40phox*, *Nox2*, *Nox4*, *KLF2*, and *p21* were also significantly high in the lymph node of Mdr2^−/−^ mice compared to the control (FVB) mice. Data are represented as mean ± SD, *n* ≥ 3. One-way ANOVA followed by Fisher LSD was used for multiple comparison. *, **, ***, **** indicates *p* ≤ 0.05, *p* ≤ 0.01, *p* ≤ 0.001, *p* ≤ 0.0001, respectively, as compared to respective control.

Recent studies indicate that the cellular metabolism plays a pivotal role in forming new lymphatic vessels [42]. Hence, we also determined the role of conjugated BAs on the cellular metabolism because LECs acquire the energy for their proliferation, sprouting, and migration from the cellular metabolic processes [43] and it is not known if conjugated BAs regulate any of these mechanisms in the LECs. To assess the role of conjugated BAs in the regulation of the LEC metabolism, we performed a Seahorse metabolic rate assay with HLECs treated with TCA (100 µM) and TCDCA (100 µM) for 24 h to measure the extracellular acidification rate (ECAR) and oxygen consumption rate (OCR) (Figure 4A–F). Both the two conjugated BAs significantly increased the ECAR in HLECs compared to the control (*p* < 0.001 and *p* < 0.01, respectively), which indicates an increase in cellular glycolysis. Both conjugated BAs increased the OCR or the mitochondrial respiration rate in HLECs (*p* < 0.05 and *p* < 0.01, respectively). Agreeing with these results, we also found that TCA and TCDCA increased the overall ATP production rate in conjugated BA-treated HLECs, and the glycolytic ATP production rate was higher compared to the control (*p* < 0.0001) (Figure 4E) and was confirmed by the ratio of the mitoATP to glycoATP production rate (*p* < 0.001) (Figure 4F). Thus, conjugated BAs may play a crucial regulatory role in the HLEC metabolism. We also analyzed the effect of conjugated BAs on the critical metabolic genes. We found that both the two conjugated BAs significantly increased the expression of the phosphofructokinase, platelet (*PFKP*), and fatty acid synthase (*FASN*) (*p* < 0.05) (Figure 4G). TCDCA showed a significant increase and TCA showed a trend towards an increase in the cytochrome c oxidase subunit I (*CO I*). The TCA also increased the expression of hexokinase 2 (*HK2*) compared to the control (Figure 4G).

It has been reported that pathological BAs activate oxidative stress in colon cancer cells and hepatocytes [44,45]. This mechanism remains completely unknown in the context of HLECs. Thus, to evaluate whether conjugated BAs induce oxidative stress in HLECs, we determined the expression of the critical regulators of the redox pathway in conjugated BA-treated HLEC. Interestingly, both TCA and TCDCA significantly increased the expression of Ras-related C3 botulinum toxin substrate 1 (*RAC1*), NADPH oxidase 4 (*Nox4*), peroxisome proliferator-activated receptor gamma (*PPARγ*), *p21CIP*, Krüppel-like factor 2 (*KLF2*), and endothelial nitric oxide synthase (*eNOS*) (Figure 4H). To corroborate our findings in vivo, we also determined the expression of the redox pathway-specific genes such as *RAC1*, *Nox1*, *Nox2* and *Nox4*, *p21*, *eNOS*, *KLF2*, *p22phox*, *p40phox*, and *p67phox* in the lymph nodes from the Mdr2^−/−^ mice. We found a significant elevation in *KLF2*, *p21*, *Nox2*, and *Nox4* and its activation subunit *p40phox* in the Mdr2^−/−^ mice lymph node compared to the WT control FVB mice (Figure 4J). We also verified that the conjugated BA-induced effects on the HLECs are mediated through the TGR5 receptor as we found that the well-known TGR5 receptor agonist significantly increased the expression of the similar set of genes (*FGFR2*, *VEGFR3*, *Prox1*, *p21CIP*, *NOX4*, and *PPARγ*) (*p* < 0.05) activated by conjugated BA (Figure 4I). 

### 3.7. TCA Enhanced the Cellular Reactive Oxygen Species (ROS) Production and Induces Prox1 SUMOylation via p90RSK Activation and Promotes VEGFR3 Expression

**Figure 5:**
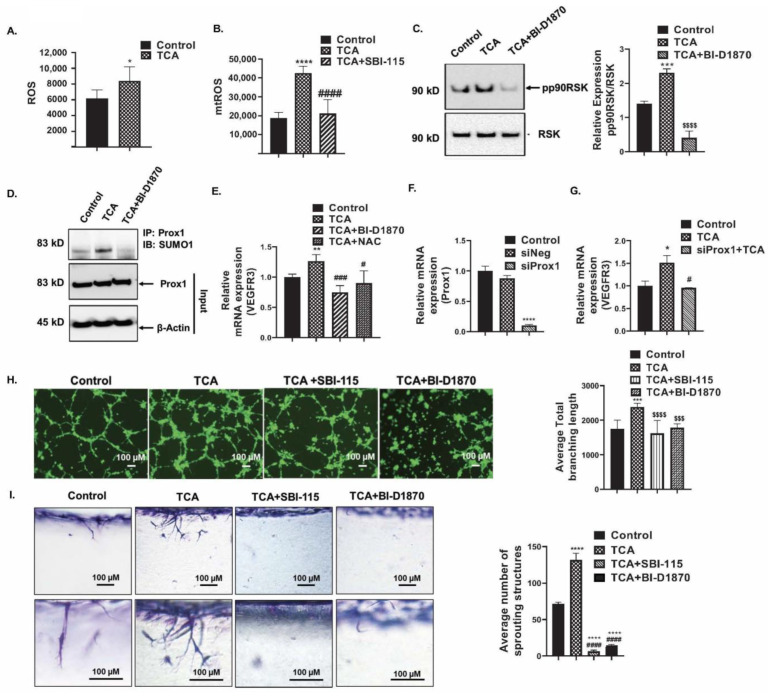
Conjugated BAs promote ROS production and induces p90RSK-mediated Prox1 SUMOylation and enhances VEGFR3. TCA (100 µM) induced (**A**) cellular ROS production at 45 min and (**B**) mtROS production after 12 h of treatment in HLEC, measured by fluorometric assay in plate reader. TGR5 antagonist SBI-115 (10 µM) pre-treatment for 1 h inhibited the TCA-induced mtROS production. (**C**) Western blot analysis showed that TCA (100 µM)-induced p90RSK phosphorylation in HLECs after 24 h, which was inhibited by p90RSK inhibitor BI-D1870. (**D**) After 24 h treatment with TCA (100 µM), the Prox1 was immunoprecipitated using anti-Prox1 antibody and probed with anti-SUMO1 antibody. TCA treatment induced Prox1 SUMOylation in HLECs which was inhibited by inhibition of p90RSK activation by using BI-D1870. (**E**) TCA (100 µM) induced *VEGFR3* mRNA expression which was inhibited by p90RSK inhibitor BI-D1870 and ROS inhibitor NAC. (**F**) *Prox1* gene was knocked down in HLECs by siProx1 and (**G**) *Prox1* knockdown inhibited the TCA-induced *VEGFR3* expression. (**H**) TCA induced tube formation of HLECs in Matrigel at 4 h, which was inhibited by the TGR5 antagonist SBI-115, p90RSK inhibitor BI-D1870. (**I**) TCA (100 µM) induced the HLEC invasion in 3D collagen matrix at 20 h and was inhibited by BI-D1870 or TGR5 antagonist SBI-115. Values represent mean ± SD, *n* ≥ 3. Statistical analysis was done by ANOVA followed by Fishers LSD. *, **, ***, **** indicates *p* ≤ 0.05, *p* ≤ 0.01, *p* ≤ 0.001, *p* ≤ 0.0001, respectively, as compared to respective control., $$$, $$$$ indicates *p* ≤ 0.001, *p* ≤ 0.0001, respectively, as compared to TCA. #, ###, #### indicates *p* ≤ 0.05, *p* ≤ 0.001, *p* ≤ 0.0001, respectively, as compared to TCDCA.

As subcellular ROS plays an essential role in modulating the endothelial cell (EC) metabolism, proliferation, and angiogenesis [46], we evaluated if the increased expression of the redox genes due to conjugated BA exposure in the HLECs was also associated with alterations in ROS. HLEC were treated with BAs (TCA, TCDCA, 100 µM) for 45 min and the total cellular ROS was measured by a fluorometric assay (Figure 5A). The TCA significantly increased the ROS production in HLEC after 45 min. The TCA also significantly enhanced the mtROS production in HLEC at 12 h, which was entirely reverted back in the presence of the TGR5 antagonist SBI-115 (10 µM) (Figure 5B). We did not find any significant change in the cellular ROS or the mtROS levels in the TCDCA-treated HLEC (data not shown). TCA-induced ROS production was inhibited by specific ROS scavenger N-Acetyl-L-Cysteine (NAC, 10 mM) (data not shown). Since TCA showed a significant effect on the production of cellular ROS and mtROS, we determined the status of p90RSK phosphorylation. The p90RSK is a redox-sensitive kinase activated via phosphorylation at S380 in the presence of ROS [47]. Interestingly, TCA significantly increased the p90RSK phosphorylation at S380 in HLECs (Figure 5C) and the p90RSK phosphorylation was inhibited by the p90RSK-specific inhibitor BI-D1870 (1 µM) treatment. As reported previously, p90RSK phosphorylation at S380 increased the SUMOylation of several nuclear proteins [48]. The post-translational modification SUMOylation can affect the fate of a protein in different ways, such as changing its cellular localization, conformation, and making it a target for ubiquitination [49]. It has also been reported that the SUMOylation of Prox1 plays a crucial role in lymphangiogenesis [17]. Thus, we determined whether TCA-induced activated pp90RSK affects the Prox1 SUMOylation. Interestingly, TCA increased the Prox1 SUMOylation, inhibited by the specific p90RSK inhibitor BI-D1870 (Figure 5D). Since SUMOylated Prox1 has been reported to act as a transcriptional activator to increase the *VEGFR3* transcription [17], we also determined if the RSK inhibitor, which inhibits Prox1 SUMOYlation (Figure 5D), also suppressed the TCA-induced *VEGFR3* mRNA expression (Figure 5E). Interestingly, the p90RSK and ROS inhibitors significantly inhibited the TCA-induced *VEGFR3* mRNA expression in HLECs (Figure 5E). Further, we confirmed that this TCA-mediated induction of the *VEGFR3* expression is mediated through *Prox1* by knocking down Prox1 with Prox1 siRNA (Figure 5F). We found that the TCA induced the *VEGFR3* expression, which was significantly reduced in Prox1 knocked-down HLEC (Figure 5G).

### 3.8. TCA-Induced p90RSK Activation Promotes the Tube Formation and Invasion of HLECs

Since TCA induced VEGFR3, a key lymphangiogenic molecule, we wanted to examine its effects on LEC tube formation. LECs showed an enhanced network-forming ability upon exposure to TCA (Figure 5H). We then wanted to investigate if this TCA-mediated tube formation occurred by p90RSK activation. Our data showed that the TCA-induced tube formation of HLECs was significantly inhibited by the p90RSK inhibitor BI-D1870, as well as the TGR5 antagonist SBI-115 (Figure 5H). We also verified if TCA induced HLEC sprouting in 3D collagen matrices and if p90RSK plays a role in this process. We observed that the treatment of HLECs with TCA significantly increased the average number of sprouting HLEC structures at 24 h. This indicated that TCA enhanced the invasion and formation of de novo lymphatic capillary-like structures and enhanced HLEC sprouting. Further, treatment with inhibitors for either TGR5 (SBI 115) or p90RSK (BI-D1870) significantly reduced the number of sprouts formed in response to TCA in comparison with the control group (Figure 5I). Thus, our data show that TCA-induced p90RSK activation plays a crucial role in the induction Prox1-VEGFR3 axis as a precursor of lymphangiogenesis and promotes lymphatic capillary formation. 

### 3.9. TCA Induced the YAP Expression and its Nuclear Translocation Mediated by the ROS 

**Figure 6:**
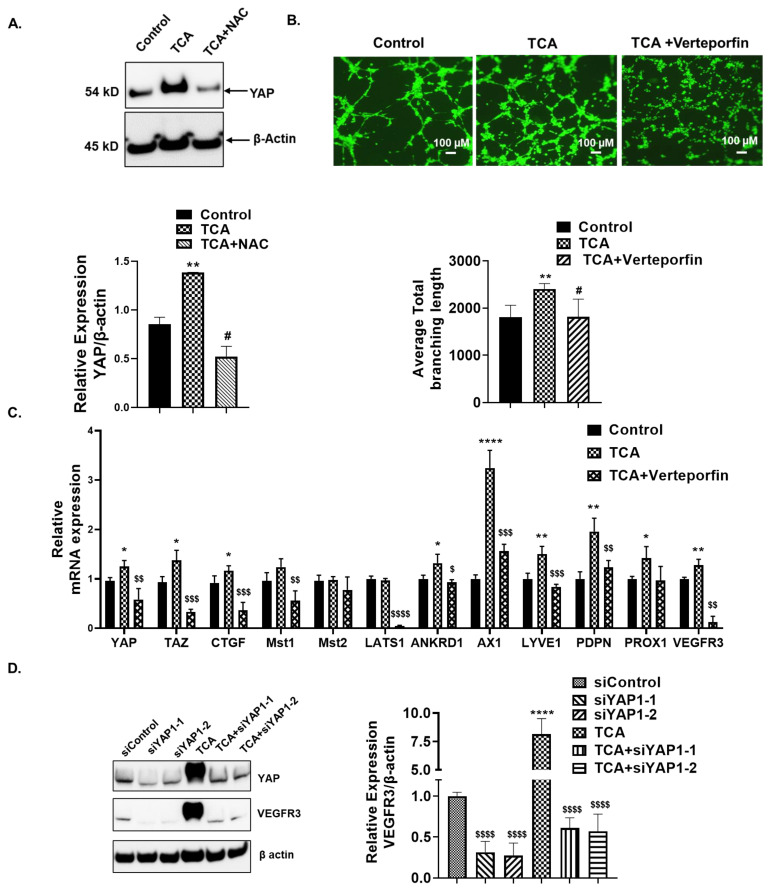
TCA-induced lymphangiogenesis of HLECs via the activation of YAP-VEGFR3 axis. (**A**) TCA (100 µM)-induced YAP expression as determined by Western blot. (**B**) YAP-TEAD inhibitor verteporfin abrogates TCA-induced tube formation in HLECs. Representative 4X images were given. Scale bar = 100 µm. Graphs represent total branching length as measured by ImageJ. Values represent mean ± SD *n* ≥ 3. Statistical analysis was done by ANOVA followed by Fishers LSD. ** Represents *p* ≤ 0.01 as compared to control. # indicates *p* ≤ 0.05 compared to TCA. (**C**) Inhibition of YAP TEAD interaction reduces TCA-mediated expression of YAP, its downstream targets, lymphangiogenic transcription factors in HLECs. Values represent mean ± SD, *n* = 3. Statistical analysis was done by ANOVA followed by Fishers LSD. *, **, and **** represents *p* ≤ 0.05, *p* ≤ 0.01 and *p* ≤ 0.0001, respectively, as compared to control. $, $$, $$$ and $$$$ represents *p* ≤ 0.05, *p* ≤ 0.01, *p* ≤ 0.001, and *p* ≤ 0.0001, respectively, compared to TCA. (**D**) Transient knockdown of YAP decreases the TCA-induced VEGFR3 expression in HLECs. A representative blot is given. Values represent mean ± SD, *n* = 3. Statistical analysis was done by ANOVA followed by Fishers LSD. **** represents *p* ≤ 0.05, *p* ≤ 0.01, *p* ≤ 0.001, and *p* ≤ 0.0001, respectively, as compared to control. $$$$ represents *p* ≤ 0.0001, respectively, compared to TCA.

YAP activation is associated with angiogenesis [50,51]. BAs have been reported to activate YAP through GPCR signaling, resulting in hepatocellular carcinoma tumorigenesis [23]. The Hippo-YAP/TAZ signaling pathway is the key player of maintaining lymphatic vascular development, regulating the LEC proliferation and migration [52]. YAP activation has also been reported to be required to maintain the lymphangiogenic transcription factor’s, PROX-1, expression during developmental lymphangiogenesis [53]. The treatment of HLECs with TCA increased the total YAP1 levels which was inhibited by the ROS inhibitor NAC (Figure 6A). 

Further, to determine the effect of YAP in lymphangiogenesis, HLECs were pretreated with the YAP-TEAD interaction inhibitor, verteporfin, and tube formation assays were performed with or without TCA. Tube formation was significantly inhibited in verteporfin + TCA groups compared to TCA (Figure 6B). Additionally, the treatment of HLECs with TCA resulted in a significant increase in the mRNA levels of *YAP* and its downstream target genes such as *CTGF*, *Axl*, and *ANKRD1* compared with the control. The verteporfin pretreatment significantly limited the observed increases in *YAP*, *TAZ*, *CTGF*, *Axl*, and *ANKRD1* (Figure 6C). Additionally, the observed upregulation in mRNA expression of lymphangiogenic genes *LYVE-1*, *PDPN*, *VEGFR3*, and *PROX1* with a conjugated BA treatment alone was significantly inhibited upon pretreatment with verteporfin (Figure 6C). 

Since our data suggested that TCA induced the activation of YAP, and subsequently lymphangiogenesis, we wanted to investigate whether the upstream regulators of this Hippo-YAP pathway were also affected. We checked the effect of conjugated BAs on key molecules of the Hippo/YAP signaling pathway, such as mammalian STE20-like 1 and 2 (*MST1/2*) and large tumor suppressor kinase 1 and 2 (*LATS1/2*). *Mst1/2* and *Lats1/2* are core kinases and play significant role in regulation and activation of YAP [54]. Upregulation of Mst1 is also directly correlated with increased severity of inflammation and hepatic injury in non-alcoholic fatty liver disease [55]. However, conjugated BAs did not alter the mRNA expression of *Mst1/2* and *Lats1* in the HLECs (Figure 6C). 

Further, to evaluate the induction of VEGFR3 by YAP in HLECs, transient knockdown with 2 different siRNA sequences targeting YAP1 was carried out. The results showed that the treatment of HLECs with TCA (Figure 6D) significantly increased the total YAP1 and lymphangiogenic growth factor receptor-VEGFR3 compared to the control. The silencing of YAP resulted in a significant reduction in the TCA-mediated increase in the YAP expression. In place of our previous data, the treatment of HLECs with TCA significantly increased the expression of *VEGFR3*. The siRNA-mediated silencing of YAP1 significantly prevented this increase in the VEGFR3 expression after the TCA treatment. Interestingly, YAP1 silencing decreased the basal expression of VEGFR3 in siControl HLECs, indicating that YAP1 is required for maintaining the expression of VEGFR3. 

## 4. Discussion

In this study, we provide a clear documentation of the expansion of the lymphatics in presclerotic liver disease and provide evidence that increased conjugated BAs activate and regulate pro-lymphangiogenic pathways. The role of conjugated BAs in liver pathologies has been investigated in several clinical studies. However, its role in the promotion of lymphangiogeneis remains unknown. Conjugated primary BAs are shown to be elevated in the serum of cirrhotic patients and considered a more sensitive biomarker of liver cirrhosis than conventional liver function tests [56]. In a urinary metabolomic study, elevated glycocholate 3-glucuronide, taurocholate, TCA, glycolithocholate 3-sulfate, and glycoursodeoxycholic acid (GUDCA) levels have been found in the serum of the Hepatitis B-infected cirrhotic patients compared to the healthy controls [57]. Further studies have also confirmed that the level of serum TCA, TCDCA, GCA, and GUDCA in cirrhotic patients strongly correlated with disease severity [58,59]. 

The liver is the largest lymph-producing organ and has a high density of lymphatics. Several studies have reported that the number of lymphatic vessels increases in the fibrotic and cirrhotic liver of humans and rats [60,61,62]. A recent study shows that chronic liver disease is associated with the increased expression of oxidized low-density lipoprotein (oxLDL) that impacts the lymphatic permeability by the VEGFR3-mediated regulation of VE-Cadherin and impedes the lymphatic transport [63]. However, the direct effects of conjugated BAs have not been evaluated on the lymphatics. It has been shown that TCA feeding increases biliary damage, liver inflammation, and fibrosis [64]. The present study aimed to explore whether the pathological BAs, e.g., TCA and TCDCA, which are elevated in cholestatic liver disease patients and cirrhotic patients, affect the lymphangiogenesis and explore the potential molecular mechanisms involved. 

In accordance with the clinical reports of the association of elevated BA levels and liver disease severity, we found a significantly high level of BAs in the serum as well as in the lymph nodes of the Mdr2^−/−^ mice, which is a well-established mouse model of fibrosing cholangiopathy, primary sclerosing cholangitis, and biliary cirrhosis [65]. Incidentally, high levels of bile acids have also been reported in metastatic nodes [6]; however, its role in pathological lymphangiogenesis remains unknown. Since hepatic lymphatics are located adjacent to bile ducts, HLECs are likely exposed to elevated levels of these pathological BAs during the onset and progression of liver disease. Our in vitro data showed that TCA and TCDCA significantly enhanced the proliferation, migration, and the tube formation ability of the HLECs, prerequisites for the progression of lymphangiogenesis. 

BAs have known target molecules for TGR5, Gpbar-1, nuclear receptors, FXR, and vitamin D receptors (VDR). Previous studies showed that the BA-induced activation of FXR leads to the ERK activation as the downstream effectors [66]. Conjugated bile acids were also reported to activate the ERK1/2 and AKT pathway in rat hepatocytes in G-protein-coupled receptors in a GPCR (G(ialpha))-dependent way via the activation of ROS [67]. BAs have been reported to activate the production of mtROS in rat hepatocytes via the ERK1/2 pathway [68]. Pathological BAs, in particular TCA, have been reported to be elevated in the serum of cholestatic liver disease patients and induce the production of ROS in human hepatocytes [69]. We have found that conjugated BAs produce ROS and mtROS in lymphatic endothelial cells. In Endothelial cells (Ecs), the cellular nicotinamide adenine dinucleotide phosphate oxidase (NOX) enzyme is the major source of cellular ROS [46]. NADPH has major subunits, namely p47phox, p67phox, p40phox, Rac1, Nox2, and p22phox. The cytosolic accessory proteins, p47phox, p67phox, and Rac1, after stimulation combined with the membrane-bound catalytic subunit Nox and p22phox [70,71]. Among the membrane-bound NOX (NADPH oxidase), multiple subunits contribute to the production of ROS [46]. The NOX-induced ROS also plays an important role in the VEGF signaling in EC [72,73,74,75]. In a recent study, Wang et al. reported a direct role of Nox4 in the lymphangiogenesis of LECs via the ROS/ERK/CCL21 pathway, which attracted CCR7-positive breast cancer cells to enter the lymphatic node [76]. Our results showed that conjugated BAs increased the expression of RAC1 and Nox4 in HLECs in vitro, which could explain conjugated BAs-induced ROS production in those cells. Interestingly, the Mdr2^−/−^ mice had elevated levels of BAs in their serum and lymph nodes. At the same time, they had also an increased expression of Nox2, Nox4, and p40phox in their lymph nodes. 

We then asked whether this conjugated BA-induced ROS targeted downstream effectors to induce lymphangiogenesis. The p90RSK is a potential downstream target of ROS in endothelial cells [19] and the activation of p90RSK via its phosphorylation is associated with EC inflammation [18]. We have found that Bas significantly increased the phosphorylation of p90RSK at S380. Heo et al. [18] reported that the activation of p90RSK under atherogenic disturbed flow phosphorylates the downstream deSUMOylating enzyme sentrin/SUMO-specific protease 2 (SENP2) at T368. Due to the phosphorylation of SENP2 at T368, SENP2 is translocated from the nucleus to cytoplasm, and as the result of this translocation, nuclear proteins such as p53 ERK5 become SUMOylated [18]. In lymphangiogenesis, Prox1 SUMOylation plays a crucial role in lymphatic development and lymphangiogenesis [17]. Prox1 is the homeodomain transcription factor that regulates the transcription of the VEGFR3, one of the most critical lymphangiogenic growth factor receptors [17], and Prox1 was identified as a target of the small ubiquitin-like modifier 1 (SUMO-1). The SUMOylation of Prox1 increased its DNA binding activity and transcriptional activity [17]. The K556 SUMOylation site on Prox1 is located within the DNA binding domain and could potentially alter its DNA binding ability and efficiency as a transcriptional activator [3]. This SUMOylation of Prox1 also reduced its interaction with histone deacetylase 3 and decreased its corepressor activity [77]. Based on these reports of the role of Prox1 SUMOylation in lymphangiogenesis, we measured Prox1 SUMOylation in conjugated BA-treated LECs in vitro and, interestingly, conjugated BAs induced Prox1 SUMOylation, which was abrogated by using the p90RSK inhibitor BI-D1870. The p90RSK inhibitor also blocked the conjugated BAs-induced VEGFR3 transcription and the lymphatic sprout formation in a 3D collagen matrix. Thus, these findings showed that conjugated BAs induced the p90RSK-Prox1SUMOylation axis as a novel pathway of the BA-induced lymphangiogenesis. The current study revealed a novel mechanism of conjugated BA-mediated lymphangiogenesis via the activation of the p90RSK-Prox1 SUMOylation axis. 

Lymphangiogenesis consists of multiple steps, including proliferation and sprouting, and is an energetically expensive process [78]. Previous reports suggest that HLECs rely on the cellular glycolysis process to acquire energy [79]. We observed that TCA- and TCDCA-treated LECs had an elevated ECAR or glycolytic rate. Importantly, our findings indicate that conjugated BAs play a critical metabolic regulatory role in LECs based on an increased rate of mitochondrial respiration in conjugated BA-treated LECs. Consequently, we found that an increase in glycolytic ATP led to an increase in the total cellular ATP in conjugated BA-treated LECs, indicated by the ATP production rate and low mitoATP to glycoATP production rate. Thus, conjugated BAs affect the overall energy demand while also shaping the metabolic phenotype of LECs, which may have important consequences in pathological lymphangiogenesis. 

These increased rates of crucial metabolic processes by conjugated BA in LECs were further supported by an increased level of expression of multiple critical metabolic genes in our study. The glycolysis process is tightly controlled by the enzymes, namely hexokinase (HK1–4), phosphofructokinase (PFKP), and pyruvate kinase [80]. It has been reported that the deletion of HK2 from HLEC impaired glycolysis and reduced the growth of lymphatic vasculature [79]. Notably, in HLECs, the HK2 expression has been reported to be regulated by FGF2 signaling [79]. The overexpression of FGF2 increases the HK2 expression as well as glycolysis in endothelial cells, which points to a probable link between glycolysis and lymphangiogenesis [79]. One of the most common BAs, deoxycholic acid (DCA), was found to increase the HK2 expression and in turn the cellular glycolysis in adenocarcinoma cells [81]. In this study, we found that while TCA and TCDCA increased the FGFR2 expression, TCA increased the HK2 expression in LECs, which supports the observed enhanced glycolysis in HLECs in the presence of those conjugated BAs. Another important enzyme in the glycolysis pathway is PFKP, which was increased by conjugated BAs in the HLECs. PFKP was reported to be highly expressed in different cancers, including breast cancer, clear cell renal cell carcinoma, lung cancer, and glioblastoma, and associated with the increase in glycolysis [82]. Our group recently showed that the CCA cells from orthotopic CCA mice model showed a high level of *PFKP* expression [83]. Along with glycolytic enzymes, fatty acid synthase (FASN), a key regulator of the fatty acid synthesis, was also reported to be involved in the lymphangiogenesis [84,85]. The inhibition of FASN with a specific inhibitor inhibits lymphangiogenesis and gives rise to an anti-metastatic environment [84]. In our current study, we found an increased expression of *FASN* in conjugated BA-treated HLECs, which is supported by the previous studies on the role of *FASN* on lymphangiogenesis. 

**Figure 7:**
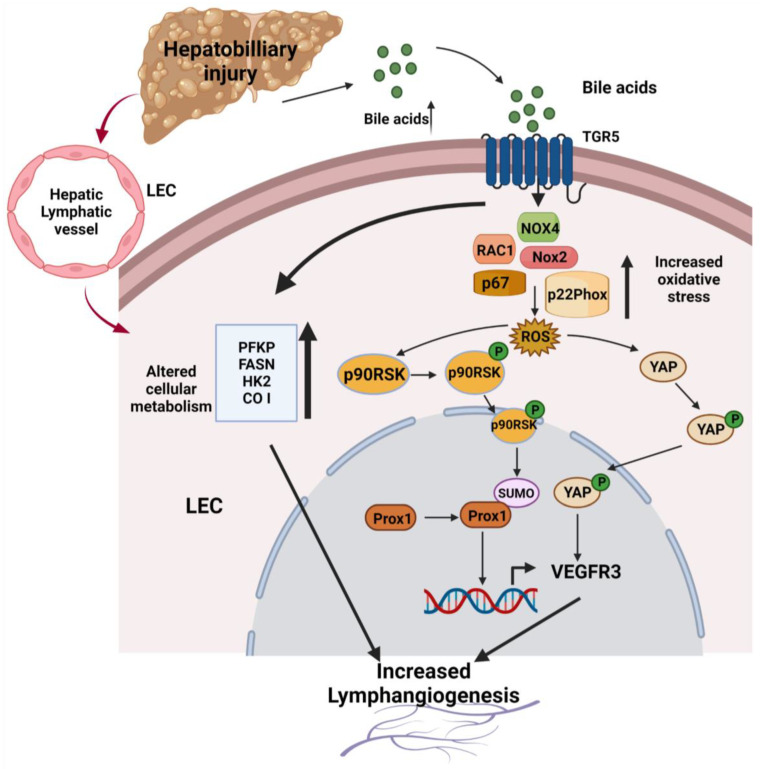
Schematic showing increased conjugated BAs induce pathological lymphangiogenesis through an ROS-p90RSK-YAP-VEGFR3-dependent mechanism. Conjugated BAs bind to TGR5 expressed by the LECs and activate several key members of the redox or oxidative stress pathway. The activation of the redox pathway in turn produces ROS and activates the p90RSK via its phosphorylation at S380. Activated pp90RSK induced the SUMOylation of Prox1, and SUMOylated Prox1 increased the transcription of VEGFR3, ultimately increasing lymphangiogenesis. Conjugated BA-induced ROS also activated the YAP, which in turn increased the lymphangiogenesis. BA also enhanced the cellular metabolism by increasing the mRNA expression of the metabolic genes, namely *PFKP*, *HK2*, *CO I*, and *FASN*, that play roles in enhancing lymphangiogenesis. Generated with BioRender.com (Toronto, ON, Canada).

This study highlighted another important axis of conjugated BA-induced lymphangiogenesis via YAP. YAP, a member of the Hippo pathway, has been previously reported to be activated in response to BAs in other cells. YAP is regulated by G-protein-coupled receptors [86] and elevated BAs levels have been shown to activate YAP in normal liver and hepatocytes isolated from liver cancer [23]. YAP acts as a transcriptional regulator of genes through coupling with the DNA-binding TEA domain family members (TEAD 1–4) after YAP is translocated to the nucleus [87]. BAs have been shown to activate YAP by dephosphorylating at S127, promoting its nuclear localization and, as a result, caused lymph node metastasis [6]. The role of YAP in the sprouting of lymphatic progenitor cells was shown in the zebrafish trunk where the paracrine VEGFC signaling induced the nuclear translocation of YAP and activated YAP [22]. Our study showed that conjugated BAs increased the expression of YAP and induced its nuclear translocation in the HLECs, which was inhibited by the ROS inhibitor NAC. Interestingly, the siRNA-mediated knockdown of cellular YAP abrogated the TCA-induced VEGFR3 upregulation, which established the BA-YAP-VEGFR3 axis as another potential lymphangiogenesis mechanism. The role of YAP in TCA-induced lymphangiogenesis was confirmed by using verteporfin, which inhibited the TCA-induced tube formation in HLEC. Verteporfin is a drug which disrupts the association between YAP and TEAD, thereby inhibiting YAP-mediated downstream effects. This inhibition of YAP-TEAD association also inhibited the TCA-induced increase in the expression of *LYVE-1*, *PDPN*, *PROX-1* as well as of the lymphangiogenic growth factor receptor *VEGFR3* in HLEC. The blocking of YAP-TEAD association also blocked the expression of the TCA-induced connective tissue growth factor (*CTGF*) in HLECs. The CTGF is involved in cell adhesion, proliferation, and angiogenesis [88]. Taken together, as shown in our pathway schematic (Figure 7), our study shows for the first time that in conjugated BA, TCA induced pathological lymphangiogenesis via inducing the oxidative stress pathways. Further, to the best of our knowledge, this is the first report of demonstrating a novel mechanism by which conjugated BAs regulate the p90RSK-YAP-VEGFR3 axis for inducing lymphangiogenesis; this signaling axis can be a potential therapeutic target for lymphangiogenesis in inflammatory pathological conditions.

## Figures and Tables

**Table 1:** Primers used in qRT-PCR.

Primers	Species	Forward	Reverse
*ANKR1*	Human	5′-AGACTCCTTCAGCCAACATGATG-3′	5′-CTCTCCATCTCTGAAATCCTCAGG-3′
*AX1*	Human	5′-AACCTTCAACTCCTGCCTTCTCG-3′	5′-CAGCTTCTCCTTCAGCTCTTCAC-3′
*CTGF*	Human	5′-AATGCTGCGAGGAGTGGGT-3′	5′-CGGCTCTAATCATAGTTGGGTCT-3′
*eNOS*	Human	5′-GAAGGCGACAATCCTGTATGGC-3′	5′-TGTTCGAGGGACACCACGTCAT-3′
*FGFR1*	Human	5′-TTAATAGCTCGGATGCGGAG-3′	5′-ACGCAGACTGGTTAGCTTCAATG-3′
*FGFR2*	Human	5′-GATAAATACTTCCAATGCAGAAGTGCT-3′	5′-TGCCCTATATAATTGGAGACCTTACA-3′
*FGFR4*	Human	5′-AGCACCCTACTGGACACACC-3′	5′-ACGCTCTCCATCACGAGACT-3′
*KLF2*	Human	5′-CCAAGAGTTCGCATCTGAAGGC-3′	5′-CCGTGTGCTTTCGGTAGTGGC-3′
*Lyve-1*	Human	5′-AGCCTGCGAAAGCCTTTTGGTG-3′	5′-GGCTTCACATTCAGCAAACCTGG-3′
*Nox4*	Human	5′-CTGCTGACGTTGCATGTTTC-3′	5′-TTCTGAGAGCTGGTTCGGTT-3′
*PDPN*	Human	5′-GTGCCGAAGATGATGTGGTGAC-3′	5′-GGACTGTGCTTTCTGAAGTTGGC-3′
*Prox-1*	Human	5′-CTGAAGACCTACTTCTCCGACG-3′	5′-GATGGCTTGACGTGCGTACTTC-3′
*PPARg*	Human	5′-AGCCTGCGAAAGCCTTTTGGTG-3′	5′-GGCTTCACATTCAGCAAACCTGG-3′
*p21CIP*	Human	5′-GGACAGCAGAGGAAGACCATGT-3′	5′-TGGAGTGGTAGAAATCTGTCATGC-3′
*RAC1*	Human	5′-GCGTTGCCATTGAACTCACC-3′	5′-GAGCTGCTACGCTCACTCCATTAC-3′
*RPL19*	Human	5′-GGGCATAGGTAAGCGGAAGG-3′	5′-TCAGGTACAGGCTGTGATACA-3′
*Ubiquitin*	Human	5′-AGTCCCTTCTCGGCGATTCT-3′	5′-GCATTGTCAAGTGACGATCACAGC-3′
*VEGFC*	Human	5′-TTCCTGCCGATGCATGTCTAA-3′	5′-TGTTCGCTGCCTGACACTGT-3′
*VEGFR1*	Human	5′-CTGCCACTCTAATTGTCAATGTGAA-3′	5′-AAACGATGACACGGCCTTTT-3′
*VEGFR2*	Human	5′-CCAGCAAAAGCAGGGAGTCTGT-3′	5′-TGTCTGTGTCATCGGAGTGATATCC-3′
*VEGFR3*	Human	5′-CCTGAAGAAGATCGCTGTTC-3′	5′-GAGAGCTGGTTCCTGGAGAT-3′
*YAP1*	Human	5′-TGTCCCAGATGAACGTCACAGC-3′	5′-TGGTGGCTGTTTCACTGGAGCA-3′
*PFKP*	Human	5′-CGGAAGTTCCTGGAGCACCTCTC-3′	5′-AAGTACACCTTGGCCCCCACGTA-3′
*HK2*	Human	5′-GAGCCACCACTCACCCTACT-3′	5′-CCAGGCATTCGGCAATGTG-3′
*CO I*	Human	5′-CTCTTGCGGTACTCATTGAAG-3′	5′-GAGCTGCTGTTCGGTGTC-3′
*GLUT3*	Human	5′-ACTTTGACGGACAAGGGAAATG-3′	5′-ACCAGTGACAGCCAACAGG-3′
*ATP6*	Human	5′-GAAGCGCCACCCTAGCAATA-3′	5′-GCTTGGATTAAGGCGACAGC-3′
*AMPK1a*	Human	5′-TGCGTGTACGAAGGAAGAATCC-3′	5′-TGTGACTTCCAGGTCTTGGAGTT-3′
*FASN*	Human	5′-CGCGTGGCCGGCTACTCCTAC-3′	5′-CGGCTGCCACACGCTCCTCT-3′
*eNOS*	Mouse	5′-TCCGGAAGGCGTTTGATC-3′	5′-GCCAAATGTGCTGGTCACC-3′
*KLF2*	Mouse	5′-CACCTAAAGGCGCATCTGCGTA-3′	5′-GTGACCTGTGTGCTTTCGGTAG-3′
*Nox1*	Mouse	5′-AATGCCCAGGATCGAGGT-3′	5′-GATGGAAGCAAAGGGAGTGA-3′
*Nox2*	Mouse	5′-CCCTTTGGTACAGCCAGTGAAGAT-3′	5′-CAATCCCGGCTCCCACTAACATCA-3′
*Nox4*	Mouse	5′-GGATCACAGAAGGTCCCTAGCAG-3′	5′-GCGGCTACATGCACACCTGAGAA-3′
*p22phox*	Mouse	5′-ATGGGGCAGATCGAGTGGGCCATGT-3′	5′-ATAGATCACACTGGCAATGGCCAA-3′
*p40phox*	Mouse	5′-GCTTCACCAGCCACTTTGTT-3′	5′-TCTTGTTTTGCGCCCATGTA-3′
*p67phox*	Mouse	5′-CCACTCGAGGATTTGCTTCA-3′	5′-ATCTTGGAATGCCTGGGCTC-3′
*p21*	Mouse	5′-CGAGAACGGTGGAACTTTGAC-3′	5′-CAGGGCTCAGGTAGACCTTG-3′
*RPL19*	Mouse	5′-ATGAGTATGCTCAGGCTACAGA-3′	5′-GCATTGGCCGATTTCATTGGTC-3′
*Ubiquitin*	Mouse	5′-GCCCAGTGTTACCACCAAGAAG-3′	5′-GCTCTTTTTAGATACTGTGGTGAGGAA-3′

**Table 2:** Antibodies used in Western blot, immunohistochemistry, and immunofluorescence.

Name of the Antibodies	Assays	Dilution	Manufacturer	Catalog Number	RRID
Phospho-p90RSK (Ser380)	WB	1:1000	Cell Signaling Technology	12032	AB_2797804
RSK1/RSK2/RSK3	WB	1:1000	Cell Signaling Technology	9355	AB_659900
Flt4/VEGFR3	WB	1:400	Santa Cruz	SC321	AB_2105107
Prox1	IP, WB	1:1000	Cell Signaling Technology	14963	AB_2783562
Lyve1	WB	1:1000			
YAP1	WB	1:1000	Novus	NB110-58358	AB_922796
pYAP Y357	IF	1:100	Sigma Aldrich	Y4645	AB_1080624
SUMO1	IP, WB	1:1000	Cell Signaling Technology	4940	AB_2302825
GPCR TGR5	IF, WB	1:100, 1:1000	Abcam	ab72608	AB_2112165
S1PR2	IF, WB	1:100, 1:1000	Thermo Fisher Scientific	PA523208	AB_2540734
FXR	IF, WB	1:100, 1:1000	Thermo Fisher Scientific	417200	AB_2532196
Beta actin	WB	1:20,000	Sigma Aldrich	A3854	AB_262011

## Data Availability

The data that support the findings of this study are available in the methods and/or available from the corresponding author on request.
